# Patient preferences for palliative treatment of locally advanced or metastatic gastric cancer and adenocarcinoma of the gastroesophageal junction: a choice-based conjoint analysis study from Germany

**DOI:** 10.1186/s12885-016-2975-9

**Published:** 2016-12-06

**Authors:** R. Hofheinz, J. Clouth, J. Borchardt-Wagner, U. Wagner, E. Weidling, M. H. Jen, P. Brück

**Affiliations:** 1 Department of Oncology, University Hospital Mannheim, Mannheim, Germany; 2 Lilly Deutschland GmbH, Bad Homburg, Germany; 3 Eli Lilly UK, Windlesham Surrey, UK; 4 Tagestherapie Zentrum am Interdisziplinären Tumorzentrum Mannheim, Universitätsmedizin Mannheim, Universität Heidelberg, Theodor-Kutzer Ufer 1-3, 68167 Mannheim, Germany

**Keywords:** Gastric cancer, Palliative chemotherapy, Conjoint analysis, Patient preferences

## Abstract

**Background:**

Decisions on palliative chemotherapy (CT) for locally advanced or metastatic gastric cancer (mGC) require trade-offs between potential benefits and risks for patients. Healthcare providers and payers agree that patient-preferences should be considered. We conducted a choice-based conjoint (CBC) analysis study in pre-treated patients from Germany with mGC or locally advanced or metastatic adenocarcinoma of the gastroesophageal junction (mGEJ-Ca), to evaluate their preferences when hypothetically selecting a CT regimen.

**Methods:**

German oncologists and gastroenterologists were contacted to identify patients with mGC or mGEJ-Ca who had completed ≥2 cycles of palliative CT in first or later lines of therapy (CT ongoing or complete). The primary objective was to quantify patient preferences for palliative CT by CBC analysis. Six in-depth qualitative interviews identified 3 attributes: treatment tolerability, quality of life in terms of ability of self-care, and additional survival benefit. The CBC matrix was constructed with 4 factor levels per attribute and each participant was presented with 15 different iterations of these levels. A minimum of 50 participants was needed. Consenting patients completed the CBC survey, choosing systematically among profiles. CBC models were estimated by multinomial logistic regression (MLR) and hierarchical Bayesian (HB) analysis. Estimates of importance for each attribute and factor-level were calculated.

**Results:**

Fifty-five patients participated in the CBC survey (78.2% male, median age 63 years, 81.8% currently receiving CT). Across this sample, low treatment toxicity was ranked highest (44.6% relative importance, MLR analysis), followed by ability to self-care (32.3%), and an additional survival benefit of up to 3 months (3 months 23.1%, 2 months 18.3%, 1 month 11.2%). The MLR analysis showed high validity (certainty 37.9%, chi square *p* < 0.01, root-likelihood 0.505). The HB analysis yielded similar results.

**Conclusions:**

Patients’ preferences related to a new hypothetical palliative CT of mGC or mGEJ-Ca can be assessed by CBCanalysis. Although in real-life, patients initially need to decide on CT before they have any experience, and patients’ varied experiences with CT will have impacted specific responses, low toxicity and self-care ability were considered as most important by this group of patients with mGC or mGEJ-Ca.

## Background

In 2012, gastric cancer remained the third most common cause of cancer death worldwide [1]. Eastern Asia, Eastern Europe, and South America are areas with a high incidence [2]. In the United States, 22,220 new cases and 10,990 cancer deaths were predicted for 2014 [3]. In Germany, an incidence of approximately 15,000 new cases was predicted for 2014, and the current 5-year survival rate is 33% [4]. These data include tumors of the gastroesophageal junction which are becoming increasingly common [5].

At the time of diagnosis, approximately 50% of patients with gastric cancer already have overt metastatic disease and are no longer eligible for a curative surgical treatment approach; chemotherapy (CT) is the mainstay of palliation and prolonging survival in this setting [5–8]. In older randomized trials evaluating the impact of adding first line CT to best supportive care, patients’ median overall survival improved from 3 months to 6 months with a combination of older CT regimens plus best supportive care. Today, patients would have to choose between a median life expectancy of 3 months with best supportive care alone and a median life expectancy of 10–12 months with a modern CT regimen [7–10].

CT for esophagogastric adenocarcinomas remains complex with varying standards of care across the world [2]. CT, with or without addition of targeted therapies, is considered the standard of care for medically fit patients, and has been associated with a survival benefit over supportive care only [2]. Treatment decisions concerning the best approaches to prolong life and preserve or improve quality of life with CT therefore require a careful trade-off between potential benefits and risks for each individual patient based on disease characteristics and comorbidities. However, the weighting of treatment goals by experts is not necessarily congruent with the preferences of affected patients [11]. Patients have to make the decision to have or not to have life-prolonging palliative CT based on the probabilities derived from research in large populations, with no personal experience of the potential benefits or toxicities of CT. Furthermore, patients have to decide which regimen/therapeutic intensity would be most suitable for them. Their decisions are influenced by experiences reported by others and on information conveyed by their physicians, their family and friends, the CT nurses, and increasingly from the internet. Patient preferences in studies are often assessed after patients have experienced the benefits, toxicities and outcomes of CT, while the above mentioned decisions have to be taken before such experiences were gained.

Patient-reported outcomes and patient preferences have become increasingly important in the current healthcare debate [12]. In Germany for example, the “Institut für Qualität und Wirtschaftlichkeit im Gesundheitswesen” (IQWiG) is obliged to consider the “patient benefit” as measured by accepted pharmacoeconomic standards when evaluating treatment options [13], and states that this will require a patient preference-based weighting of relevant endpoints by established methods such as conjoint analysis [14–16].

Choice-based conjoint (CBC) analysis has indeed become a well-established method to quantify patient preferences [11, 17], and has been applied successfully to measure preferences for a diverse range of health applications, including cancer treatments [17]. In contrast to other common malignancies such as lung or breast cancer, however, patient preferences for palliative CT of locally advanced or metastatic gastric cancer (mGC) have not been evaluated so far, neither in Germany nor in any international studies [17]. Due to the specific clinical situation of these patients, such as their specific problems associated with food intake, ascites, or maldigestion, patient preferences may differ from those identified for the treatment of other malignancies on the attribute level as well as on the weighing of different attributes.

Therefore, we conducted the current study to assess patient preferences for a new hypothetical palliative CT of gastric cancer in Germany, using a CBC analysis approach, in patients with previous or ongoing CT exposure. We interviewed 55 patients with mGC or locally advanced or metastatic adenocarcinomas of the gastroesophageal junction (mGEJ-Ca) who had received at least 2 cycles of palliative CT in first or later lines of therapy. The purpose was (1) to evaluate if CBC analysis can be used appropriately in this type of severely ill cancer patients, and (2) to quantify patients’ preferences for palliative CT when they need to trade-off between different attributes while comparing them to direct patients’ treatment goals.

## Methods

The study and all interviews were conducted in accordance with guidelines published by the European Pharmaceutical Market Research Association (EphMRA) and the European Society for Opinion and Marketing Research (ESOMAR) [18, 19]. Hospital- and practice-based oncologists and gastroenterologists throughout Germany involved in gastric cancer treatment were contacted and asked to identify patients who met the target criteria and were willing to participate in the study.

Both qualitative and quantitative interviews were conducted predominantly at the patients’ homes, or at any location preferred by the patient. Selected moderators with several years of experience in pharmaceutical and patient market research conducted the interviews; all patients had the option to be accompanied by a trusted person throughout the interview. Both the qualitative and the quantitative surveys were conducted by MaritzCX, Hamburg, Germany.

### Study sample

The target population consisted of adult patients (≥18 years) with cytologically or histologically confirmed diagnosis of mGC or mGEJ-Ca who had received at least 2 cycles of palliative CT in first or later lines of therapy. This CT could either be ongoing or have been completed within the last 2 years. Patients had to be physically and mentally capable to participate in a 45-60 min interview as per opinion of the treating physician.

Patients were recruited by their treating physicians. Eligible patients received a written patient information sheet from their physicians which contained the project description and a response sheet. Patients willing to participate were asked to send the response sheet to the research agency, and the interview was then set up.

### Qualitative in-depth interviews

Interviewers experienced in quantitative and qualitative healthcare research projects (MaritzCX, Hamburg, Germany) conducted 6 initial in-depth interviews. The interviews were designed to identify those aspects that patients considered as particularly relevant for palliative care of their gastric cancer. Patients’ general experience with the disease, perceived limitations in the daily routine and in coping with them, perceived benefits and limitations associated with gastric cancer treatment, and the patients’ attitudes towards treatment, treatment needs and treatment goals were addressed.

The interviews were taped, analyzed, and the information collected was used to develop the quantitative survey described below, and to define the attributes and attribute factor levels for the CBC matrix as outlined below.

### Quantitative interviews

#### Direct questioning

During the 55 quantitative interviews, a programmed questionnaire was used by the trained interviewers for data collection; all data were collected in a pseudonymized format. Data collected included demographic data and key disease characteristics, weight loss, and a rating of overall perceived capabilities. Experience with CT was categorized as currently receiving or not, but no further details were collected. In addition, patients’ were directly asked which treatment goal they considered as most important and what additional goals they had for the treatment of their mGC (open-ended questions). Finally, patients were asked to rate the extent of disease-related limitations regarding their preferred activities, eating, social activities with friends, family and partner relationships, and self-care during daily living, on a Likert scale ranging from 1 (very mild) to 5 (very strong). In addition, they were asked to name any other perceived limitations of their activities during daily living they associated with their disease. The interviewer entered all responses directly into a tablet or laptop PC during the interview. No additional patient data were collected from other sources, e.g. the treating physicians.

#### Conjoint analysis module

The qualitative in-depth interviews formed the basis for the development of the CBC analysis matrix that assessed patient preferences for a new hypothetical palliative CT of gastric cancer [20]. The matrix spanned 3 attributes including “ability to self-care” as a measure for performance status and quality of life, and “treatment tolerability”, and “additional survival benefit” as key attributes. These 3 attributes had been identified as most relevant for patients during the qualitative interviews. In order to keep the quantitative interviews manageable even for severely ill participants, the CBC matrix was kept as simple as possible, with only 3 attributes and 4 different factor levels each (Table 1), and the number of choice tasks for each patient (iterations) was limited to 15. The levels were chosen for each attribute in such a way that the difference between levels would be reasonable and easily understandable for the participants as well as medically sound, e.g. the additional survival benefit should reflect the differences seen in median overall survival between older doublet and more modern triplet regimens.

**Table 1:** Attributes and levels used for the choice-based conjoint analysis

Attributes		Factor levels			
		Level 1	Level 2	Level 3	Level 4
1.	Ability to self-care	No assistance required for activities of daily living	Little assistance required for activities of daily living	A lot of assistance required for activities of daily living	Complete assistance required for activities of daily living; bed-ridden
2.	Treatment tolerability (adverse reactions)	No or mild adverse reactions possible; no hospitalization required	Moderate adverse reactions possible; manageable without hospitalization	Severe adverse reactions possible; hospitalization for 3–4 days may be required	Very severe to life-threatening adverse reactions possible; hospitalization for ≥5 days may be required
3.	Survival benefit (vs. standard of care)	No additional survival benefit	Survival benefit of approximately 1 additional month	Survival benefit of approximately 2 additional months	Survival benefit of approximately 3 additional months

During each of the 15 choice tasks, the patient had to choose which treatment he would prefer among 3 hypothetical treatment profiles with different factor levels for each of the 3 attributes. The interviewer presented these hypothetical treatment profiles to the patient on the screen of a tablet or laptop PC, and then entered the choices into the tablet or laptop PC on the patient’s behalf.

### Statistical analysis

#### Sample size

Sample size considerations were based on the standard formula for sample size estimation for CBC analysis published by Johnson and Orme. A minimum sample size of 50 patients was required for the planned CBC design (3 attributes with 4 factor levels each, 15 iterations) [21].

#### Direct questioning

All data collected by direct questioning (demographic data, disease characteristics, perceived disease-related limitations, treatment goals) were evaluated descriptively.

#### Conjoint analysis

Results of the conjoint analysis models were estimated by a) aggregate multinomial logistic regression (MLR) [20] and b) hierarchical Bayesian (HB) analysis [20, 22]. MLR modelling mainly describes the preference at the group level rather than at the individual patient level, while HB estimation additionally considers patterns at the individual patient level. The Sawtooth Software packages SMRT 4.20 and CBC/HB 4.6.4 (Sawtooth Software, Inc., Orem, Utah, USA) were used for the MLR and HB analyses, respectively [20].

The validity of the MLR approach was evaluated based on the percent certainty (likelihood ratio-index) and chi square statistics. Both models were evaluated by the root likelihood. The root likelihood is an intuitive measure of how well the solution fits the data. The best possible value is 1.0 (indicating perfect model fit), and the worst possible value is the reciprocal of the number of choices available in the average task, i.e. 0.33 in this study (indicating no model fit) [20]. In addition, the validity of both models was checked by repeating all analyses in 2 subgroups derived from a randomly generated 50:50 sample split. The robustness of the model could be confirmed if the root likelihood values were similar for the overall sample and the 2 subgroups.

Estimates of relative importance were calculated for each factor level (part-worth utilities) and aggregated for each attribute (total utility). The part-worth utilities were scaled and normalized in a way that the lowest factor level for each attribute was assigned a part-worth utility of 0, and the combination of the best factor levels for all 3 attributes resulted in a total utility of 100. The relative importance of an individual attribute thus corresponds to the difference between the highest and the lowest (0) standardized part-worth utility for that attribute.

## Results

### Qualitative interviews

The qualitative interviews (*N* = 6) revealed that patients with mGC or mGEJ-Ca who had at least some experience with CT did not evaluate their palliative CT for gastric cancer based on the survival benefit per se, but rather on the extent of survival benefit associated with a high perceived quality of life, which they predominantly characterized as being able to self-care and receiving a CT with good tolerability. Therefore, the three aspects “ability to self-care”, “treatment tolerability” and “survival benefit” were the key factors used to define the 3 attributes for the CBC matrix (Table 1).

### Quantitative phase - direct questioning

**Fig. 1:**
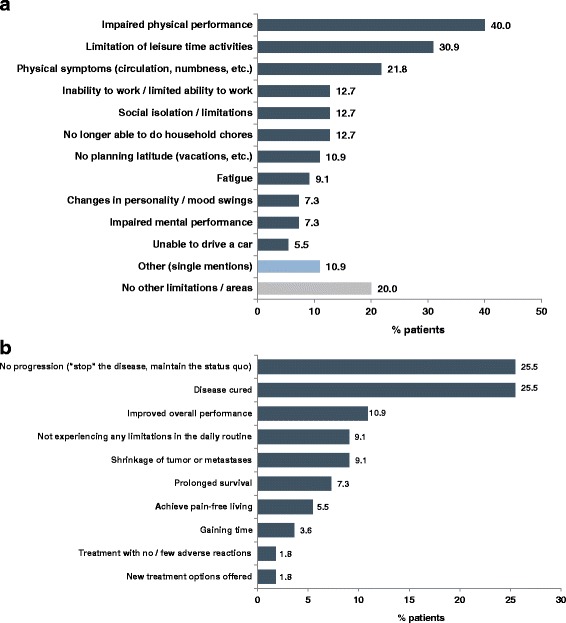
Direct, open-ended questioning: Summary of perceived disease-related limitations (Panel **a**) and treatment goals (Panel **b**) (*N* = 55). **a**. Perceived disease-related limitations (open-ended question, multiple responses possible). **b**. Most important treatment goals (single responses only). Abbreviations: N, number of patients in the study sample

A total of 55 additional patients with mGC or mGEJ-Ca participated in the quantitative survey (face to face interviews by trained personnel), 78.2% male, median age 63 years; Table 2). More than 80% of these 55 patients were receiving CT at the time the interview was conducted (81.8%, Table 2), and the majority felt their perceived capabilities were much worse than before diagnosis (65.5%). On average, patients perceived disease-related limitations to be most pronounced for their preferred activities (mean index score 3.3, score ranged from 1 [very weak] to 5 [very strong]), eating (3.1), and social activities with friends (2.8). Family and partner relationships (2.2) and self-care during daily living (2.0) were perceived as less affected. The most common disease-related limitations that the patients specified by additional open-ended questioning included physical limitations (40.0%), limitations of leisure time activities (30.9%), and physical symptoms (21.8%) (Fig. 1a).

**Table 2:** Baseline characteristics (*N* = 55)

Characteristic	
Age [years]	
Median (range)	63 (42–85)
≥ 65 years, n (%)	25 (45.5)
Sex, n (%)	
Male	43 (78.2)
Female	12 (21.8)
Relationship, n (%)	
Married	39 (70.9)
Single	9 (16.4)
Domestic partnership	5 (9.1)
Widowed	2 (3.6)
Children yes/no	44/11 (80.0/20.0)
Living area, n (%)	
Large city (≥100,000 residents)	28 (50.9)
Rural area or small city (<20,000 residents)	15 (27.3)
Medium-sized city (20,000 to <100,000 residents)	12 (21.8)
Current performance, change versus performance before diagnosis, n (%)	
Much worse	36 (65.5)
Slightly worse	17 (30.9)
Unchanged	2 (3.6)
Improved	0
Weight loss during the last 6 months, n (%)	
Pronounced (>3 kg)	36 (65.5)
Mild (2–3 kg)	8 (14.5)
None	11 (20.0)
Gastric resection, n (%)	
Complete resection	18 (32.7)
Partial resection	12 (21.8)
No resection	25 (45.4)
Currently receiving chemotherapy, n (%)	45 (81.8)

*n* number of patients, *N* number of patients in study sample

When questioned directly, one fourth of the patients each stated that their most important treatment goal was to avoid disease progression (25.5%) or to achieve cure of the disease (25.5%), respectively, followed by improved overall performance (10.9%; Fig. 1b). The most commonly reported additional treatment goal (multiple responses possible) was “to experience no limitations in daily routine” (27.3%). Adding up related treatment goals showed that improving survival (cure, prolonged survival, or gaining time) was the most important goal for 54.6% of patients; avoiding disease progression or achieving tumor shrinkage was most important for 34.6% of patients, while treatment goals related to symptom improvement (improved overall performance, no limitations in daily routine, and pain-free living) were most important for 25.5% of patients.

### Conjoint analysis

**Fig. 2:**
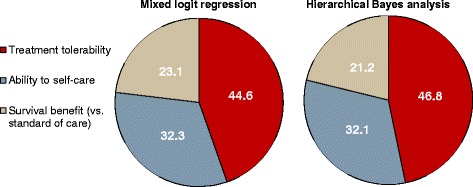
Conjoint Analysis: Relative importance of the 3 attributes (*N* = 55), analyzed by multinomial logistic regression (MLR, left pie) and hierarchical Bayesian analysis (HB, right pie)

**Fig. 3:**
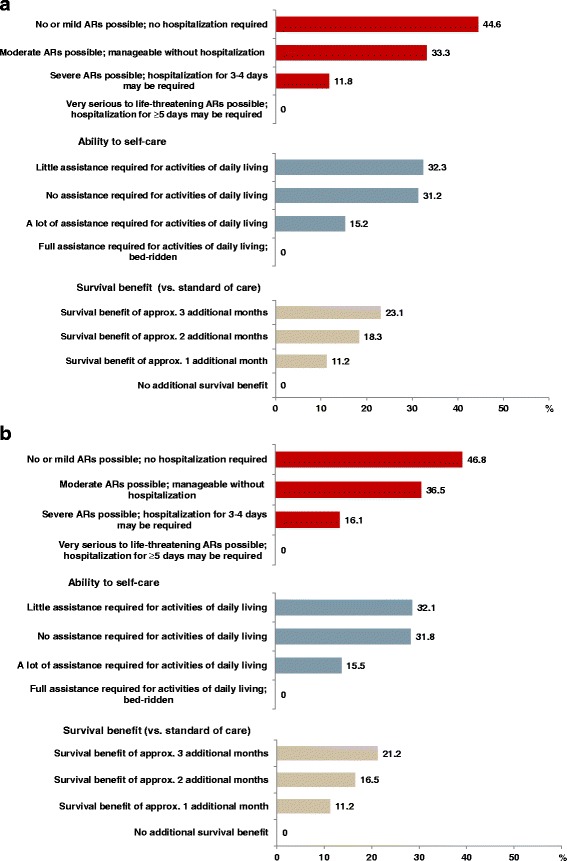
Conjoint analysis: Relative importance of the individual factor levels (*N* = 55), analyzed by multinomial logistic regression (MLR, panel **a**) and hierarchical Bayesian analysis (HB, panel **b**). **a** Multinomial logistic regression analysis. **b** Hierarchical Bayesian analysis. Abbreviations: approx., approximately; AR, adverse reactions; N, number of patients in study sample

All 55 patients completed the conjoint analysis; 51 (92.7%) perceived the complete survey, including the conjoint analysis, as positive or very positive. The 2 different modeling approaches for the data, MLR and HB, both indicated that the models had high validity (MLR: certainty 37.9%, chi square *p* < 0.01, root likelihood 0.505; HB: root likelihood 0.732). In addition, both analyses gave consistent results, in the overall sample (Figs. 2 and 3) as well as in 2 subgroups generated by a random 50:50 split of the overall sample (data not shown).

Based on both MLR and HB modeling (Fig. 2a and b), patients considered low treatment toxicity as the most important preference (relative importance: MLR 44.6%, HB 46.8%), followed by ability to self-care (MLR 32.3%, HB 32.1%), and an additional survival benefit of up to 3 months (MLR: 3 months 23.1%, 2 months 18.3%, 1 month 11.2%).

Patients valued a treatment associated with no or mild adverse reactions only and requiring no hospitalization twice as important (MLR 1.93fold, HB 2.21fold as important) as a survival benefit of 3 additional months over standard of care (relative importance: MLR 44.6% vs. 23.1%, Fig. 3a; HB 46.8% vs. 21.2%, Fig. 3b). Also, they considered requiring “little assistance for activities of daily living” 1.4-1.5times as important as a survival benefit of 3 additional months over standard of care (relative importance: MLR 32.3% vs. 23.1%, Fig. 3a; HB 32.1% vs. 21.2%, Fig. 3b).

## Discussion

To our knowledge, this study provides the first patient preference data for a new hypothetical palliative CT of gastric cancer, performed after patients had started treatment. All patients were able to complete the CBC module, and most (92.7%) perceived the complete survey as a positive or very positive experience, confirming that CBC analysis can be appropriately used in these severely ill patients. In this CBC analysis, treatment tolerability and the ability to self-care were ranked highest in importance by a sample of 55 patients with mGC or mGEJ-Ca and varied CT experience over the last 2 years. A palliative CT associated with no or mild adverse reactions and requiring no hospitalization was considered twice as important as an additional 3-month survival benefit, and requiring little or no assistance for daily living activities was considered 1.5 times as important as an additional 3-month survival benefit. The findings indicate that patients with previous CT experience consider a survival benefit accompanied by high quality of life, i.e. being able to self-care and receiving a treatment with good tolerability, as more important than an additional survival benefit per se. In direct questioning, the importance of survival was perceived higher than in the CBC analysis, yet the weighted responses of patients trading off between different aspects of their daily life, disease and treatment in the CBC model provide a broader picture and should therefore be considered as more complete when evaluating patient preferences. In the end, this interpretation is consistent with the results of the 6 qualitative interviews, and with the results from direct, open-ended questioning, where goals related to prolonged survival (prolonged survival, cure, or gaining time) were most frequently mentioned as the most important treatment goals, followed by avoiding disease progression or achieving tumor shrinkage, and treatment goals related to symptom improvement (improved overall performance, no limitations in daily routine, pain-free living). Nevertheless, physicians should be aware that they need to word their questions carefully when trying to identify their patients’ true preferences. Patient preferences may have differed depending on patients’ main treatment goals. However, the sample size (*N* = 55) precluded any subgroup analysis by treatment goal.

Patient preferences have been previously evaluated for other tumor entities such as breast cancer or non-small cell lung cancer (NSCLC) [23, 24]. These studies indicate that preferences may differ considerably, depending on factors such as tumor type, severity of disease, and extent of previous treatment. For example, a CBC study in 121 patients with Stage I-IV breast cancer, all treated with CT during the last 5 years, identified a survival benefit of 3 months as the most important preference. These patients considered a more convenient administration regimen as less important than a 13% chance or more of severe toxicities, but more important than a 10–12% chance of severe toxicities [23]. In another recent study, 211 patients with NSCLC who had been treated within the last 2 years considered an increase in progression-free survival as the most important factor, followed by a reduction in tumor-associated symptoms (cough, shortness of breath, and pain), and the reduction of side effects. Mode of administration was considered as least important.

Subgroup analyses revealed that the relative importance of “progression-free survival” increased with therapy experience [24].

In all these previous investigations, as well as the current study, patients were already exposed to CT before patient preferences were assessed. In this study, more than 80% of patients were currently receiving CT when they completed the survey. This limits the informative value for the strategic decision for or against CT based on median survival data from randomized clinical trials as patients still do not have any experience of the potential benefits and toxicities. Yet, the results give hints for patients’ preferences when choosing between different treatment options. Also, patients who have previously experienced a palliative benefit (e.g. improved dysphagia) or tumor response can be expected to be more in favor of CT than patients who had progressive disease and experienced adverse reactions. In addition, untreated, less severely ill patients might consider adverse reactions as less important and survival benefit as more important than patients currently suffering from adverse reactions during CT. Because performance status, the treatment regimen given, the timing of the survey in relation to patients’ ongoing CT (i.e. during recovery period between cycles or during acute toxicity phase), tumor response, and toxicity data were not captured in this survey, their impact on patient preferences could not be assessed. This might be considered as considerable limitation. On the other hand, including different patients with different CT experiences may help to mirror the real-life situation more closely. Further, the study included only patients who were willing to participate and were considered fit enough for participation by their physician. Therefore, the study population may not be representative of the general population of gastric cancer patients receiving palliative CT.

Another limitation of the study is that while the maximum additional survival benefit over standard of care in the fictive patient profiles of the conjoint analysis survey was 3 months which reflects the differences between various modern CT regimens (older vs. more modern, doublet vs. triplet), modern first-line CT regimens offer a more pronounced survival benefit of up to 9 months over best supportive care [7–10]. Finally, the sample size was limited in our study, and it cannot be excluded that the recruitment procedure (treating physicians contacted target patients) may have resulted in selection bias. On the other hand, the high root likelihood values for both models (MLR and HB) and the consistency of results across different model approaches indicate that the CBC analysis provided high-validity results.

## Conclusions

Patient preferences related to a hypothetical new palliative CT of gastric cancer can be assessed by CBC analysis performed after patients have gained at least some experience of their own toxicity profile and the effect of CT on their cancer. Though patients’ varied experiences with CT will have impacted specific responses, across this sample of patients with esophagogastric adenocarcinoma, low toxicity and self-care ability were ranked highest in importance. These preferences of patients already under CT might not reflect the actual preferences of all patients with mGC and mGEJ-Ca, but may nevertheless help to guide the strategic decision between different CT regimens of so far untreated patients, as well as those faced with the decision about subsequent therapy. Future studies will have to validate this approach by gaining more detailed and real-world evidence, i.e. by evaluating patient preferences before and during CT exposure in a longitudinal study, considering the impact of tumor response on patient preferences.

## Abbreviations

CBC: Conjoint-based analysis
CT: Chemotherapy
GC: Gastric cancer
HB: Hierarchical Bayesian analysis
IQWiG: Institut für Qualität und Wirtschaftlichkeit im Gesundheitswesen
mGC: Locally advanced or metastatic gastric cancer
mGEJ-Ca: Locally advanced or metastatic adenocarcinoma of the gastroesophageal junction metastatic adenocarcinoma of the gastroesophageal junction
MLR: Multinomial logistic regression
NSCLC: Non-small cell lung cancer
